# Functionalization of niobium nitrogen-doped titanium dioxide (TiO_2_) nanoparticles with ethanolic extract*s* of *Mentha arvensis*

**DOI:** 10.1186/s11671-024-04011-x

**Published:** 2024-04-15

**Authors:** Muhammad Awais Farooqi, Hafiz Muhammad Umer Farooqi, Theophilus Bhatti, Ghayas Uddin Siddiqui, Farzana Kausar, Chul Ung Kang

**Affiliations:** 1 Department of Mechatronics Engineering, Jeju National University, Jeju-si, Republic of Korea; 2 Board of Governors Regenerative Medicine Institute, Cedars-Sinai Medical Center, Los Angeles, CA USA; 3 Interdisciplinary Department of Advanced Convergence Technology and Science, College of Pharmacy, Jeju National University, Jeju-si, Republic of Korea; 4 Department of Chemical and Biological Engineering, Jeju National University, Jeju-si, Republic of Korea; 5 Department of Plant Sciences, Quaid-i-Azam University, Islamabad, Pakistan

**Keywords:** Nb-N-TiO_2_, *Mentha arvensis*, DPPH, FRAP, SEM, FTIR, Antioxidant activity

## Abstract

Titanium dioxide (TiO_2_) nanoparticles have gained significant attention due to their wide-ranging applications. This research explores an approach to functionalize Niobium Nitrogen Titanium Dioxide nanoparticles (Nb-N-TiO_2_ NPs) with *Mentha arvensis* ethanolic leaf extracts. This functionalization allows doped NPs to interact with the bioactive compounds in extracts, synergizing their antioxidant activity. While previous studies have investigated the antioxidant properties of TiO_2_ NPs synthesized using ethanolic extracts of *Mentha arvensis*, limited research has focused on evaluating the antioxidant potential of doped nanoparticles functionalized with plant extracts. The characterization analyses are employed by Fourier-transform infrared spectroscopy (FTIR), scanning electron microscopy (SEM), and Ultraviolet–visible (UV–Vis) spectroscopy to evaluate these functionalized doped nanoparticles thoroughly. Subsequently, the antioxidant capabilities through the 2,2-diphenyl-1-picrylhydrazyl (DPPH) and ferric-reducing antioxidant power (FRAP) assays have been assessed. Within functionalized Nb-N-TiO_2_, the FTIR has a distinctive peak at 2350, 2010, 1312, 1212, and 1010 cm^−1^ with decreased transmittance associated with vibrations linked to the Nb-N bond. SEM revealed a triangular aggregation pattern, 500 nm to 2 µm of functionalized Nb-N-TiO_2_ NPs. Functionalized doped Nb-N-TiO_2_ NPs at 500 µg mL^−1^ exhibited particularly robust antioxidant activity, achieving an impressive 79% efficacy at DPPH assessment; meanwhile, ferric reduction efficiency of functionalized doped Nb-N-TiO_2_ showed maximum 72.16%. In conclusion, doped Nb-N-TiO_2_ NPs exhibit significantly enhanced antioxidant properties when functionalized with *Mentha arvensis* ethanolic extract compared to pure Nb-N-TiO_2_ manifested that doped Nb-N-TiO_2_ have broad promising endeavors for various biomedicine applications.

## Introduction

Nanotechnology, an ever-evolving frontier within science and technology, is dedicated to fabricating materials at the nano-scale, offering many applications in synthetic and biological chemistry [1]. Nanostructures, or nanoparticles, showcase a distinct array of physical, chemical, and biological attributes dictated by their dimensions, geometries, and morphologies [2]. While various conventional methods exist for synthesizing nanoparticles, concerns regarding their environmental concerns, cost-effectiveness, and toxicity have driven sustainable alternatives [3]. Enter the "Functionalization Nanotechnology" domain, a visionary approach using botanical extracts and fruit-derived solutions to functionalize nanoparticles [4]. Metal oxide NPs, a burgeoning category of materials, have recently garnered substantial attention owing to their potential contributions to scientific research and health-related advancements [5]. Among these, titanium dioxide (TiO_2_) NPs have emerged as remarkable due to their inherent antioxidant properties [6]. However, TiO_2_ NPs' allure extends beyond antioxidants, as they have been exhaustively scrutinized for their capability to augment various properties, predominantly because of their distinctive electronic and catalytic attributes [7]. Meanwhile, introducing niobium and nitrogen dopants into titanium dioxide transforms its crystal structure, engendering localized defects and supplementary energy levels within the bandgap [8]. These energy levels expedite the transfer of electrons, resulting in enhanced electron mobility and superior electron transfer rates compared to their pristine TiO_2_ counterparts [9]. Some recent studies have utilized metal and organic wastes as potential raw materials for producing PNC-doped TiO2 NPs. The analysis confirmed the incorporation of nitrogen, phosphorus, and carbon-related species during synthesis. Upon integration into water-based polymeric paints, these NPs demonstrated significant photocatalytic activity, effectively removing 96% of surface-adsorbed pollutants under natural sunlight and UV radiation [10]. Recent advancements in functionalizing titanium dioxide (TiO2) nanoparticles (NPs) for biomedical applications have focused on enhancing their biocompatibility, targeting capabilities, and therapeutic efficacy [11]. Surface functionalization of TiO2 NPs with biocompatible coatings, such as polyethylene glycol (PEG) or other polymers, has been explored to minimize cytotoxicity and enhance their compatibility with biological systems [12]. These coatings provide a protective layer around the NPs, reducing their interactions with biological components and improving their overall biocompatibility [13]. Functionalized TiO2 NPs have been designed to deliver therapeutic agents, such as anticancer drugs, to specific target sites in the body [14]. Surface modifications with targeting ligands, such as antibodies or peptides, enable selective binding to receptors overexpressed on cancer cells, enhancing the accumulation of NPs at tumor sites and minimizing off-target effects [15]. TiO_2_ NPs functionalized with photosensitizing molecules have been investigated for photodynamic therapy, a non-invasive treatment modality for cancer [16]. Upon irradiation with light of a specific wavelength, these NPs generate reactive oxygen species (ROS), leading to localized cell death and tumor regression [12]. Surface functionalization allows for the controlled release of photosensitizers and enhanced tumor targeting [17]. Functionalized TiO_2_ NPs have been utilized as contrast agents for various imaging modalities, including magnetic resonance imaging (MRI), computed tomography (CT), and photoacoustic imaging. Surface modifications with imaging probes or contrast agents enable specific targeting of diseased tissues or organs, improving the sensitivity and specificity of diagnostic imaging [18]. Functionalized TiO_2_ NPs with dual functionality for therapy and diagnostics, known as theranostic NPs, have emerged as promising tools for personalized medicine [15]. These NPs can simultaneously deliver therapeutic agents while providing real-time imaging feedback on treatment efficacy, allowing for personalized treatment strategies and monitoring of disease progression [18].

Furthermore, the doping process remodels the nanoparticles' surface morphology, thereby increasing their surface area and furnishing additional active sites for catalytic reactions [19]. The intense redox potential of niobium nitrogen-doped titanium dioxide NPs renders them highly efficient in the antioxidation of reactive oxygen species (ROS) [20]. It can be helpful in health remediation applications. The amalgamation of doped NP attributes and phytochemical constituents may yield a superlative antioxidant capacity [21]. For instance, the ethanolic leaf extracts of *M. arvensis* harbor an array of phytochemicals, including rosmarinic acid, tannins, vitamin C, and various other bioactive compounds, all renowned for their antioxidant prowess [22]. These phytochemicals act as effective scavengers of ROS and mitigators of oxidative stress [23]. When these phytochemicals interface with the functionalized nanoparticles, they amplify their antioxidant capacity. Functionalizing nanoparticles with *M. arvensis* ethanolic leaf extracts engenders an expansion of the surface area, providing a more extensive landscape for phytochemical-nanoparticle interactions. This enlarged surface area creates additional reaction sites for ROS neutralization, further enhancing antioxidant activity [24].

Moreover, Niobium Nitrogen-Doped Titanium Dioxide NPs, distinguished for their exceptional electron transfer capabilities, harmonize effortlessly with *M. arvensis* ethanolic leaf extracts to engender heightened redox reactions, augmenting ROS scavenging and bolstering antioxidant activity [25]. *M. arvensis* ethanolic leaf extracts are intrinsically safe, biocompatible, and environmentally benign, rendering them ideal candidates for doped nanoparticle functionalization in biomedical applications [26]. This symbiosis between *M. arvensis* ethanolic leaf extracts and doped nanoparticles Nb-N-TiO_2_ minimizes the risk of toxicity, thus positioning these hybrids as promising contenders for antioxidant therapies within biomedicine and pharmaceuticals [27]. Leveraging *M. arvensis* ethanolic leaf extracts as doped nanoparticle functionalization agents epitomizes the "Functionalization" approach. This methodology offers many advantages, including cost-effectiveness and scalability, while diminishing the necessity for hazardous chemicals [28]. However, it is imperative to note that research about the antioxidant properties of doped TiO_2_ nanoparticles functionalized with *M. arvensis* ethanolic leaf extracts remains in its nascent stages [29]. Consequently, this study is devoted to elucidating the functionalization of Niobium Nitrogen-Doped Titanium Dioxide (TiO_2_) NPs by using *M. arvensis* ethanolic leaf extracts and delving into their antioxidant potential [30]. This endeavor aspires to contribute to our comprehension of the antioxidant attributes of Niobium Nitrogen-Doped Titanium Dioxide (TiO_2_) NPs functionalized with *M. arvensis* ethanolic leaf extracts and their potential as a pioneering avenue for combatting oxidative stress [31].

The presented work explores the novel approach of functionalizing Niobium Nitrogen-Doped Titanium Dioxide (Nb-N-TiO2) NPs with ethanolic extracts of Mentha arvensis leaves. While previous research has examined the antioxidant properties of TiO2 nanoparticles synthesized using Mentha arvensis extracts, more attention must be given to evaluating the antioxidant potential of doped NPs functionalized with plant extracts [7]. Through comprehensive characterization analyses employing Fourier-transform infrared spectroscopy (FTIR), scanning electron microscopy (SEM), and Ultraviolet–visible (UV–Vis) spectroscopy, the study thoroughly assesses the functionalized doped nanoparticles. The antioxidant capabilities of the functionalized Nb-N-TiO_2_ are evaluated using 2,2-diphenyl-1-picrylhydrazyl (DPPH) and ferric-reducing antioxidant power (FRAP) assays [32]. The results demonstrate that functionalized doped Nb-N-TiO_2_ nanoparticles exhibit significantly enhanced antioxidant properties compared to pure Nb-N-TiO_2_, highlighting their potential for various biomedical applications. This research represents a novel advancement in nanotechnology and biofunctional materials, offering promising avenues for further exploration and application [29].

## Methods

### Synthesis of niobium and nitrogen co-doped titanium dioxide (Nb-N-TiO_2_) NPs

The sol–gel methodology was harnessed to fabricate niobium and nitrogen-co-doped titanium dioxide, denoted as Nb-N-TiO_2_ nanoparticles [33]. Following the protocol, a 500 mL glass vessel was utilized to add 20 mL of a 97% titanium tetraisopropoxide (TTIP) solution with 150 mL of 2-propanol [34]. Subsequently, a gradual addition of 20 mL of TTIP while maintaining continuous agitation for 15 min [35]. Following this, 6 mL of acetic acid was introduced and stirred for 30 min, culminating in a heated environment until the temperature equilibrated at 70 °C [36]. Upon achieving this thermal threshold, 6 mL of ethylene glycol was methodically incorporated, and agitation continued without additional heat for 20 min [37]. A urea solution was methodically introduced to this synthesized solution and underwent stirring for an additional 20 min. Subsequently, the solution harboring the niobium precursor was added and subjected to an extended stirring regimen of 45 min [38]. It is imperative to emphasize that rigorous agitation was diligently maintained throughout the procedure to ensure homogeneous mixing and proper integration of the components [39]. The resultant solution, emblematic of meticulous precision, was permitted to undergo a maturation process spanning 20 h. After that, it was subjected to a controlled drying process at 100 °C for 24 h. The subsequent phases of the fabrication process encompassed the comminution of the material, followed by a controlled calcination process at 450 °C for a comprehensive duration of 4 h [39]. This elevated temperature calcination process effectively removed most organic impurities, resulting in a measurable reduction in the material's overall mass. In a culminating step, the synthesized Nb-N-TiO_2_ material underwent further functionalization by incorporating ethanolic extracts from *M. arvensis* [3].

### Collection of plant material

The botanical specimen chosen for this study, *Mentha arvensis*, is a plant in the mint family Lamiaceae and was sourced from the Jeju-si, located within the Jeju-do region of the Republic of Korea. *Mentha arvensis*, the plant used in this study, was cultivated in a garden [39]. As there are no specific local or national guidelines for the cultivation or use of this plant, no permissions or licenses were required for its cultivation and use in research. The Plant Sciences Department at Jeju National University verified the authenticity of the plant species [2].The leaves were carefully harvested and underwent a rigorous cleaning process, including multiple cycles of immersion and rinsing in running and distilled water, to ensure the purity and cleanliness of the plant material [1].

### Preparation of *Mentha arvensis* leaf ethanolic extract

**Fig. 1:**
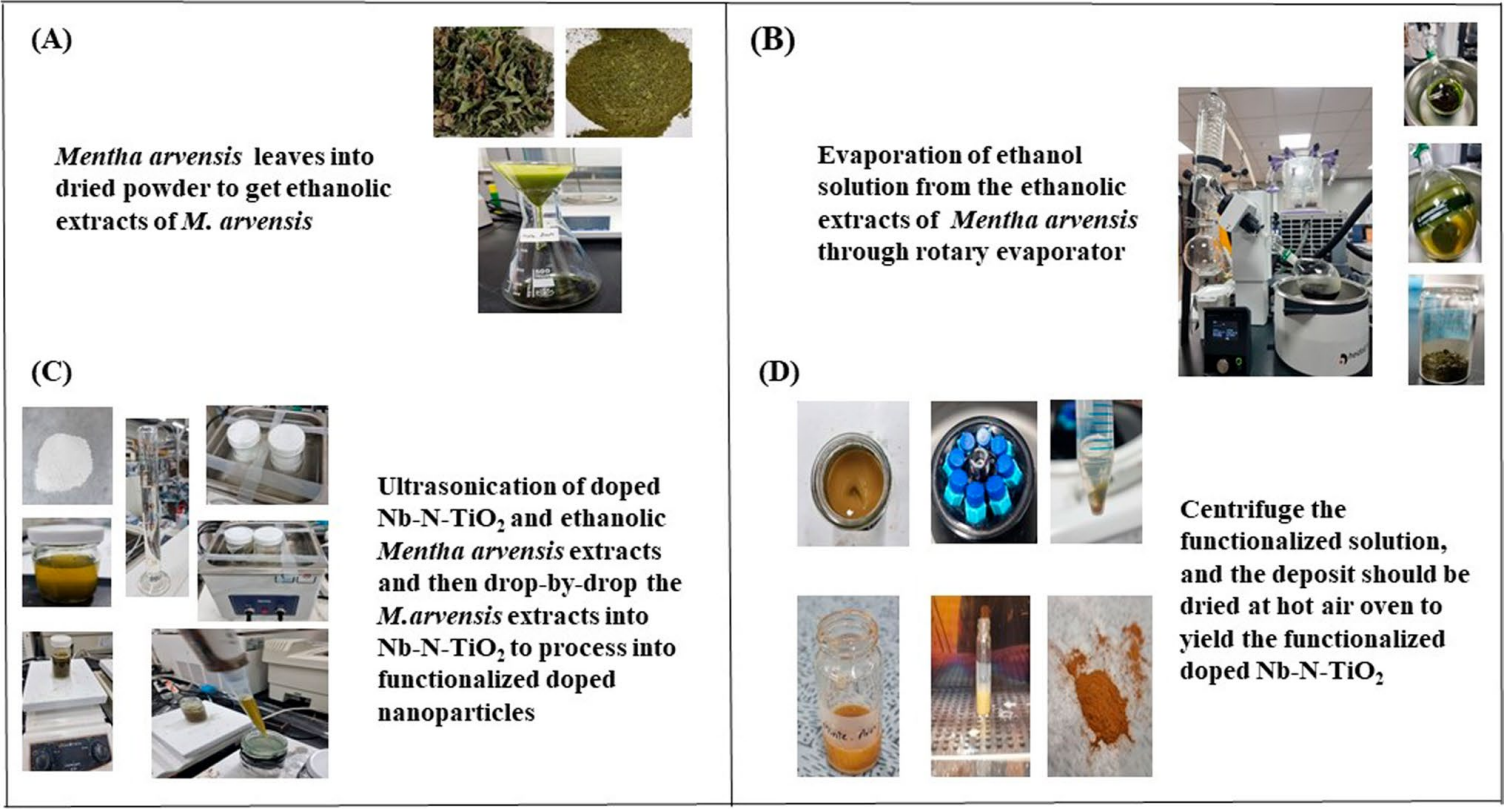
Functionalization process of doped Nb-N-TiO_2_ NPs: **A** Conversion of *M. arvensis* leaves into dried powder to obtain ethanolic extracts of *M. arvensis*, **B** Evaporation of the ethanol solution from the ethanolic extracts of *M. arvensis* through a rotary evaporator, **C** Ultrasonication of doped Nb-N-TiO_2_ and ethanolic *M. arvensis* extracts, followed by dropwise addition of *M. arvensis* extracts into Nb-N-TiO_2_ for the formation of functionalized doped NPs, **D** Centrifugation of the functionalized solution, with the resulting deposit dried in a hot air oven to yield the functionalized doped Nb-N-TiO_2_

First, the leaves were judiciously air-dried under shaded conditions at ambient room temperature for approximately seven days, ensuring optimal preservation of their inherent properties material [40]. Following this desiccation phase, the desiccated *M. arvensis* leaves underwent a fine powder using a traditional mortar and pestle technique, enabling a uniform particle-size material. 25 g of the finely powdered leaf material was combined with 250 mL of ethanol to initiate the extraction process; *Mentha arvensis* ethanolic extract was extracted precisely, employing the ultrasonication extraction technique using the PowerSonic 520 ultrasonic machine (Hiep Phat) for 3 h at 30 °C with the machine set at a high-speed level 4 [41]. The ensuing procedure spanned a continuous extraction period of 3 h, designed to maximize the extraction efficiency of bioactive constituents from the medicinal plant extracts [42]. Then, it was filtered with 20 Whatman filter paper, as shown in Fig. 1. Subsequently, the ethanol was subjected to an evaporation process by employing a rotary evaporator machine (Heidolph) with a chiller temperature of − 7 °C, water bath temperature set at 50 °C, and a rotation speed of 80 rpm to acquire a highly concentrated ethanolic *M. arvensis* extract saturated with the *M. arvensis* antioxidant potential [4]. This valuable ethanolic extract, characterized by its concentration and purity, was carefully transferred into sealed containers, safeguarding its integrity and efficacy and for further functionalization with doped Nb-N-TiO_2_ NPs [5].

### Functionalization of Nb-N-TiO_2_ NPs with ***M. arvensis***

This procedure combines 500 mg of *M. arvensis* dried extract powder in 50 mL of deionized (DI) water with a dispersion containing 1000 mg of Nb-N-TiO_2_ NPs in 50 mL of DI water [6]. The prepared *M. arvensis* extract solution is added to the doped Nb-N-TiO_2_ nanoparticle solution drop by drop, followed by continuous agitation at 500 rpm using a magnetic stirrer [7]. This continuous stirring regimen transpires at room temperature and is sustained for six hours. Notably, an observable alteration in color occurs during this stirring phase, providing insights into the transformative interaction between Nb-N-TiO_2_ NPs with *M. arvensis* ethanolic extracts [8]. Simultaneously, continuous UV–visible readings, taken at four distinct time points with two-hour intervals, serve as invaluable probes into the interaction dynamics between the ethanolic extract and the doped Nb-N-TiO_2_ NPs surface [9]. These readings interpret any alterations in the optical properties of the doped nanoparticles in real time, enabling precise absorbance measurements [19]. Upon completion of the stirring phase, the solution is centrifuged at a formidable speed of 12,000 revolutions per minute (rpm) for 15 min [20]. The supernatant is discarded, and the functionalized Nb-N-TiO_2_ NPs are isolated, undergoing a triple rinsing with DI water. The isolated functionalized Nb-N-TiO_2_ NPs are then subject to desiccation within a hot air oven, maintained at a controlled temperature of 80 °C for approximately 2 h [21]. Subsequent phases encompass the comprehensive characterization of the dried functionalized doped NPs through diverse analytical techniques, i.e., FTIR and SEM, culminating in a comprehensive understanding of their properties and potential antioxidant applications [22]. Recent studies have explored the synthesis of nanosized Co_3_O_4_ catalysts using extracts from *microalgae Spirulina platensis*, *Chlorella vulgaris*, and *Haematococcus pluvialis* to enhance carbon monoxide oxidation efficiency. Varied calcination temperatures resulted in Co_3_O_4_ catalysts with different morphologies, influenced by the presence of phosphorus and potassium from the extracts, significantly improving catalytic activity. Structural characterization techniques revealed the involvement of metabolites from the extracts in the synthesis process [43]. Additionally, research highlights the synthesis of silver, TiO_2_, cobalt (II) hydroxide, and cobalt (II, III) oxide nanomaterials from *Spirulina platensis* extract, showing antifungal activity against *Candida* species [44]. The ethanolic extracts of *M. arvensis* were tested to enhance the activity of Nb-N-TiO_2_ NPs, comprising bioactive components such as menthol, menthone, menthyl acetate, pulegone, rosmarinic acid, flavonoids, and tannins, known for their antioxidant properties. Characterization techniques like UV–Vis spectroscopy and FTIR analysis likely identified these components in the extracts before and after NP synthesis [45]. The *M. arvensis* components and Nb-N-TiO_2_ NPs interact through surface adsorption and chemical bonding, facilitated by functional groups like hydroxyl and carbonyl. This leads to surface functionalization and modulation of physicochemical properties. These interactions offer opportunities for controlled delivery of therapeutic agents or functional molecules [24].

### Statistical Analysis

All samples were analyzed in triplicate using one-way analysis of sample *t* test using Graphpad prism 8.0.1 software. (H. J. Motulsky, Prism 8 Statistics Guide, GraphPad Software Inc., San Diego, CA, USA, www.GraphPad.com). A *p*-value of < 0.05 was judged statistically significant. Graphs were created using Originpro [46].

## Characterization of Nb-N-TiO_2_ NPs

### UV–visible spectroscopy

The validation of Nb-N-TiO_2_ functionalization within the reaction blend, augmented with ethanolic *M. arvensis* extracts, was evaluated through UV–visible spectrophotometry [23]. A stringent and established protocol was adhered to for this analytical assessment [24]. The reaction between doped Nb-N-TiO_2_ and *M. arvensis* ethanolic leaf extracts was subjected to comprehensive spectral scanning across the extensive wavelength span of 200 to 700 nm [25]. This methodical analysis was executed using a sophisticated UV–visible spectrophotometer, specifically the renowned LAMBDA 850 + UV/Vis Spectrophotometer by PerkinElmer [26]. The acquired dataset encompassed the discernment and precise recording of the predominant absorbance peak, interpreting the transformation occurring within the reaction milieu [27].

### Fourier transform-infrared spectroscopy (FTIR)

The comprehension of the functional groups inherent to the botanical constituents responsible for capping, reducing, and stabilizing Nb-N-TiO_2_ was systematically achieved through Advanced, Ultra-Compact Fourier-transform infrared (FTIR) by Bruker [28]. In a summary of the methodology, the reaction blend containing the three different materials, ethanolic *M. arvensis* extracts, pure Nb-N-TiO_2_, and functionalized Nb-N-TiO_2_, was subjected to a centrifugal force of 8000 revolutions per minute (rpm), persisting for 10 min [47]. The resultant pellet was subjected to a series of thorough rinses with water, whereby the centrifugation process was iterated thrice to ensure comprehensive purification [42]. Subsequently, the acquired pellet was diligently desiccated within a controlled environment. For the ensuing analytical step, precisely 1 mg of this ethanolic plant extract, pure Nb-N-TiO_2_, and functionalized Nb-N-TiO_2_ were employed to prepare optically transparent specimen discs, this was accomplished through a coating process with 0.1 g of potassium bromide (KBr) pellets [5]. These specimen discs were then subjected to FTIR analysis, characterized by spectral scanning spanning the extensive range from 500 to 4000 cm^−1^, achieved at a resolution of 4 cm^−1^ [6]. This multifaceted analytical approach facilitated the discernment and comprehensive characterization of the crucial functional groups, underpinning the remarkable properties of ethanolic *M. arvensis* extracts, pure Nb-N-TiO_2_, and functionalized Nb-N-TiO_2_ in this designed study [19].

### Morphological analysis of Nb-N-TiO_2_ NPs

The morphological attributes, encompassing structural intricacies, geometry, and dimensional attributes, of the ethanolic *M. arvensis* extracts, pure Nb-N-TiO_2_, and functionalized Nb-N-TiO_2_ achieved through the fabrication process were investigated through Scanning Electron Microscopy (SEM). Specifically, the cutting-edge MIRA3 TESCAN SEM instrument was employed in this analytical attempt [20]. In a concise delineation of the experimental protocol, the SEM apparatus was configured to operate at an accelerating voltage of 10 kV. Subsequently, the three materials, ethanolic *M. arvensis* extracts, pure Nb-N-TiO_2_, and functionalized Nb-N-TiO_2_ nanoparticles, were judiciously and uniformly distributed onto the surface of the sample holder, respectively, following which the prepared specimen underwent a systematic examination under the SEM's discerning lens [23]. Multiple magnification levels were strategically employed during this investigation, affording a comprehensive and profound comprehension of the extracts' and doped nanoparticles' characteristics at the nanomicroscopic scale [24].

### DPPH radical scavenging activity

The evaluation of the radical scavenging prowess of ethanolic *M. arvensis* extracts, pure Nb-N-TiO_2_, and functionalized Nb-N-TiO_2_ was executed through the validated DPPH (1,1-diphenyl-2-picrylhydrazyl) radical scavenging assay, an epitome of analytical precision [25]. In the experimental protocol, 10 mL of ethanolic solution containing 0.1 mM of DPPH was mixed with 1.2 mL of various concentrations ranging from 7.8 to 500 µg mL^−1^ of the prepared plant extracts, pure Nb-N-TiO_2_, functionalized samples [26]. This solution was then subjected to an incubation period of 30 min under opaque incubator conditions. After this incubation period, a color change was observed, as the solution's color transitioned from an initial purple to a vibrant yellow, indicative of the DPPH radical scavenging activity [27]. The quantitative assessment of this transformation was evaluated through absorbance measurements at a wavelength of 519 nm, carried out by the instrumentality of a Spark multi-plate reader (Tecan) [28]. It is of notable significance to underscore that L-Ascorbic Acid (LAA) was entitled as the positive control in this antioxidant assessment, anchoring the scientific reliability and robustness of the study [29].

### FRAP ferric reducing antioxidant power assay

Evaluating the potassium ferricyanide reducing potential of ethanolic *M. arvensis* extracts, pure doped Nb-N-TiO_2_, and functionalized Nb-N-TiO_2_, was accomplished by following a standardized reducing assay protocol, with minor adaptations, reflecting the commitment to precision [30]. In this procedure, a uniform concentration was taken as standard of 500 µg mL^−1^ of *M. arvensis*, Nb-N-TiO_2_, and functionalized Nb-N-TiO_2_ was added with 2.5 mL each of 1% potassium ferricyanide (C6N6FeK3) and 200 mM phosphate buffer (pH 6.5) [29]. Subsequently, the reaction blend was subjected to an incubation phase, meticulously sustained at 55 °C for a calibrated duration of 15 min [30]. Following this thermal treatment, a rapid cooling regimen ensued, succeeded by the incorporation of 2.5 mL of 10% trichloroacetic acid (C_2_HCl_3_O_2_). The ensuing phase involved a centrifugal purification step, executed at 3,500 rpm for 12 min [31]. The ensuing pellet was systematically discarded, while the supernatant was harmoniously merged with an equal volume of double-distilled water and 0.1% (1 mL) ferric chloride (FeCl^3^). Subsequently, the absorbance magnitude was discerningly quantified at a precise wavelength of 700 nm, employing the specificity and reliability of a Spark multi-plate reader (Tecan) [48]. This methodical approach optimizes the antioxidant potential of materials, offering profound insights into the reducing potential of ethanolic *M. arvensis* extracts, pure Nb-N-TiO_2_ and functionalized Nb-N-TiO_2_ [49].

## Results

### UV–Vis spectrum analysis

**Fig. 2:**
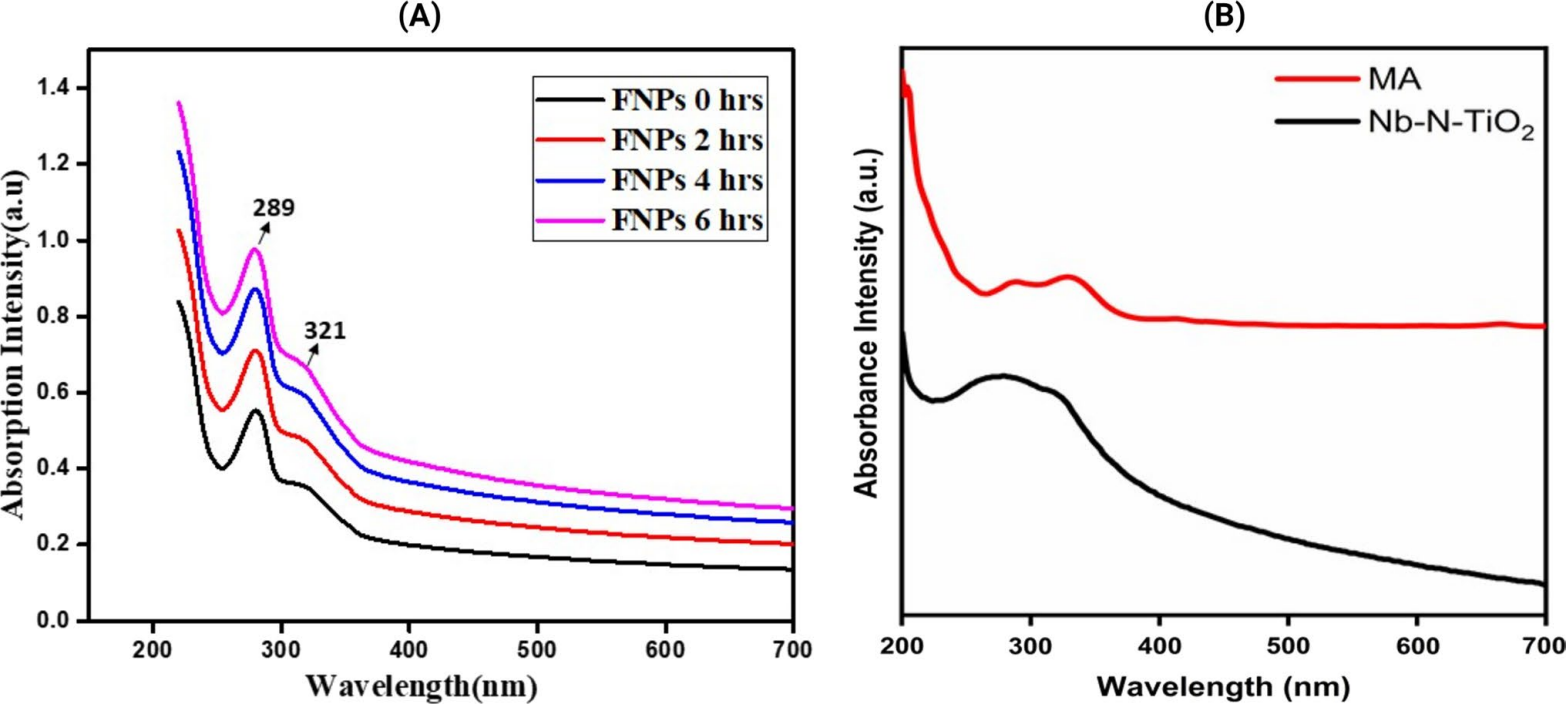
LAMBDA 850 + UV/Vis Spectrophotometer absorbance readings **A** Functionalized Nb-N-TiO_2_ with two hours’ intervals **B** *M. arvensis* ethanolic extracts and pure Nb-N-TiO_2_

The UV–visible spectrum showed absorbance peaks at 289 nm and 321 nm wavelengths. The peak at 289 nm corresponds to the absorbance of the *M. arvensis* ethanolic extracts. In comparison, the notable peak at 321 nm signifies the absorbance emanating from the functionalized doped Nb-N-TiO_2_ with *M. arvensis* ethanolic extracts, depicted in Fig. 2A and B. *M. arvensis* ethanolic extracts and pure Nb-N-TiO_2_ [50]. Simultaneously, in some previous investigations, TiO_2_ nanoparticles functionalized from ethanolic extracts of *M. arvensis* have exhibited absorbance peaks at 291 nm and 318 nm [51]. Conversely, alternative research has reported that TiO_2_ synthesized using aqueous leaf extracts of *M. arvensis* showed absorbance peaks ranging from 388 to 413 nm [52]. This variance in the observed absorbance peaks can be attributed, in part, to the presence of Niobium-nitrogen and TiO_2_ constituents, possessing an ensemble of free electrons, thus markedly contributing to the increase of the Surface Plasmon Resonance (SPR) absorption band [53]. The recent scientific literature has recurrently reported a spectrum of plant-based doped TiO_2_ functionalized modalities, each yielding absorbance peaks spanning from 380 to 450 nm [54]. Nevertheless, the optimal absorbance for plant extracts is conventionally plotted within the narrow range of 415–425 nm [3]. These notable inconsistencies in the observed absorbance peaks may be attributed to a complex interaction of multiple factors, i.e., nanoparticle size and the type, quantity, duration of functionalization, and quality of phytochemical constituents in the botanical extract [7].

### FTIR analysis

**Fig. 3:**
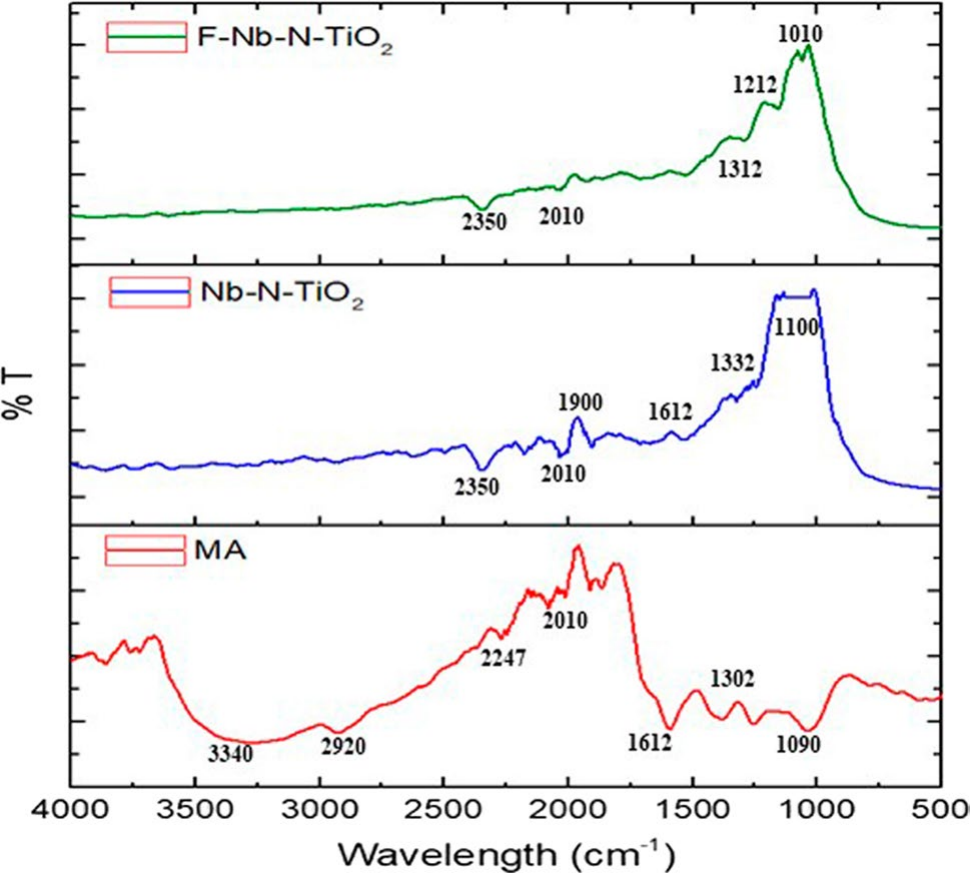
FTIR transmittance peaks of *M.arvensis* ethanolic extract (MA) extracts, Nb-N-TiO_2_, and functionalized Nb-N-TiO_2_ (F-Nb-N-TiO_2_)

The exploration of functional groups originating from the *M. arvensis* ethanolic extracts phytochemicals and the surfaces of pure doped Nb-N-TiO_2_ and functionalized Nb-N-TiO_2_ were undertaken through the prism of Fourier-transform infrared (FTIR) analysis. The results from the FTIR investigation have revealed spectral patterns with sharp and perceptible peaks, demonstrating the molecular change between the samples. The results obtained from FTIR analysis have revealed significant peaks at specific wavenumbers for the initial pure doped Nb-N-TiO_2_ nanoparticles (NPs), the pure doped Nb-N-TiO_2_NPs, and the functionalized Nb-N-TiO_2_ NPs. Notably, distinctive peaks at 3340, 2920, 2247, 2010, 1612, and 1090 cm^−1^ were observed for the initial M. arvensis ethanolic extracts, likely corresponding to O–H stretching vibrations, C-H stretching vibrations, nitrogen-containing groups, carbon-containing functional groups, C=C stretching vibrations, and C-O stretching vibrations, respectively [45]. In contrast, the pure doped Nb-N-TiO_2_ NPs exhibited distinct peaks at 2350, 2010, 1612, 1332, and 1100 cm^−1^, indicating the presence of nitrogen-containing groups, carbon-containing functional groups, C=C stretching vibrations, C–N stretching vibrations, and C–O stretching vibrations, respectively [55]. Upon functionalization, notable peaks at 2350, 2010, 1312, 1212, and 1010 cm^−1^ were elegantly observed, accompanied by a decrease in transmittance, illustrated in Fig. 3. These peaks may signify the incorporation of organic moieties from the *M. arvensis* ethanolic extracts onto the Nb-N-TiO2 NPs. The weaker intensity and smaller peaks observed in the FTIR spectra of FNb-N-TiO_2_ can be attributed to inherent differences in the organic and inorganic functional groups [3]. The intensity and peak size changes suggest variations in surface coverage and adsorption efficiency during the functionalization process, influenced by factors such as the initial concentration of *M. arvensis* ethanolic extracts and the available surface area on the Nb-N-TiO_2_ NPs [21]. Furthermore, the differences in FTIR spectra among Nb-N-TiO_2_ NPs and *M. arvensis* ethanolic extracts stem from the distinct chemical nature of each component [33]. FNb-N-TiO_2_ exhibits lower intensity during functionalization, resulting in weaker peaks influenced by factors such as functionalization reaction time, temperature, and the concentration of *M. arvensis* extracts and Nb-N-TiO_2_ NPs [36]. These observed changes in the FTIR spectra provide compelling evidence of a refined interaction between the inorganic doped NPs and organic plant extracts during the functionalization process, confirming the successful incorporation of functional groups onto the Nb-N-TiO_2_ NPs [37].

### SEM analyses

**Fig. 4:**
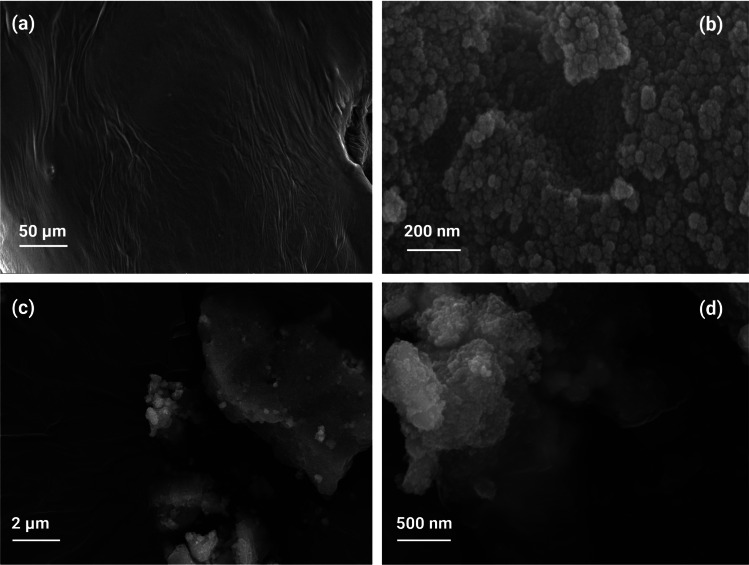
FESEM image of **a** *M. arvensis* ethanolic extracts, **b** pure doped Nb-N-TiO_2_ NPs, **c** and **d** functionalized Nb-N-TiO_2_ NPs

The examination of nanoparticle morphology stemming from *M. arvensis* ethanolic extracts, pure doped Nb-N-TiO_2_, and functionalized Nb-N-TiO_2_ was executed through the lens of scanning electron microscopy (SEM) [35]. These in-depth microscopic analyses have unveiled revelations about their structural attributes [36]. A noteworthy revelation from SEM observations is the distinct flat morphology exhibited by the *M. arvensis* ethanolic extract ranging from 50 to 100 µm [33]. In contrast, pure Nb-N-TiO_2_ showed finely spherical and triangular features, often congregating in the aggregated clusters. Nb-N-TiO_2_ NPs present a size spectrum ranging from 80.51 to 200 nm [38]. A remarkable spectacle unfolds when we focus on the functionalized Nb-N-TiO_2_ NPs, revealing a triangular aggregation pattern ranging from 500 nm to 2 µm, captured in Fig. 4 [48]. These variances in shape and size in the SEM images emphasize the profound influence of capping active phytochemicals derived from the ethanolic extracts of *M. arvensis* upon the doped Nb-N-TiO_2_ NPs morphology [55]. This symphony of diverse forms also reported previous research, where studies involving ethanolic extracts of *M. arvensis* have reported the presence of spherical and triangular-shaped TiO_2_ NPs [50].

Additionally, some recent findings depict resonance in TiO_2_ synthesis from ethanolic extracts of *M. arvensis*, where TiO_2_ NPs emerged as spherical-shaped entities embodying dimensions within the size span of 60–80 nm [31]. Further, TiO_2_ synthesized from the ethanolic leaves extract of *M. arvensis* is a captivating spherical and triangular garb, showcasing an average size of 60 nm. These SEM revelations portray a consistent trend in nanoparticle morphology, influenced by the *M. arvensis* in their functionalization with doped Nb-N-TiO_2_ [54]. This further correlates between Nb-N-TiO_2_ characteristics, the alchemical composition of *M. arvensis* phytochemicals, and the functionalization process. In this scientific exploration, these SEMs correspond with FTIR analysis and the UV–visible spectrum, culminating in the characteristics of the doped functionalized NPs properties [25]. Ethanolic *M.arvensis* extract is organic and supports doped Nb-N-TiO_2_ NPs in functionalization process. The ethanolic extract of *M.arvensis* used in functionalization comprises various organic bioactive compounds, including menthol, menthone, and menthyl acetate [45]. These organic constituents can play vital roles in interacting with the doped NPs and the overall functionalization process. Firstly, the extract's organic content provides functional groups, such as hydroxyl (− OH), carbonyl (C=O), and aromatic rings, which can facilitate the adsorption of NPs onto their surface [24]. This adsorption occurs through non-covalent interactions, including hydrogen bonding, van der Waals forces, and electrostatic interactions. The organic molecules act as anchoring points for the NPs, allowing them to adhere to the surface and preventing aggregation [32]. Moreover, the organic content may also act as a stabilizing agent for the NPs, helping maintain their dispersion and preventing agglomerating. This stabilization is crucial for ensuring the uniform distribution of NPs and optimizing their performance in various applications [6]. Additionally, the organic compounds present in the *M.arvensis* extract can influence the surface chemistry of the doped Nb-N-TiO_2_ NPs. For instance, functional groups such as hydroxyl (− OH) and carbonyl (C=O) groups can undergo chemical reactions with the surface atoms of the doped NPs, leading to surface functionalization and modification of their physicochemical properties [28].

### DPPH radical scavenging activity

**Fig. 5:**
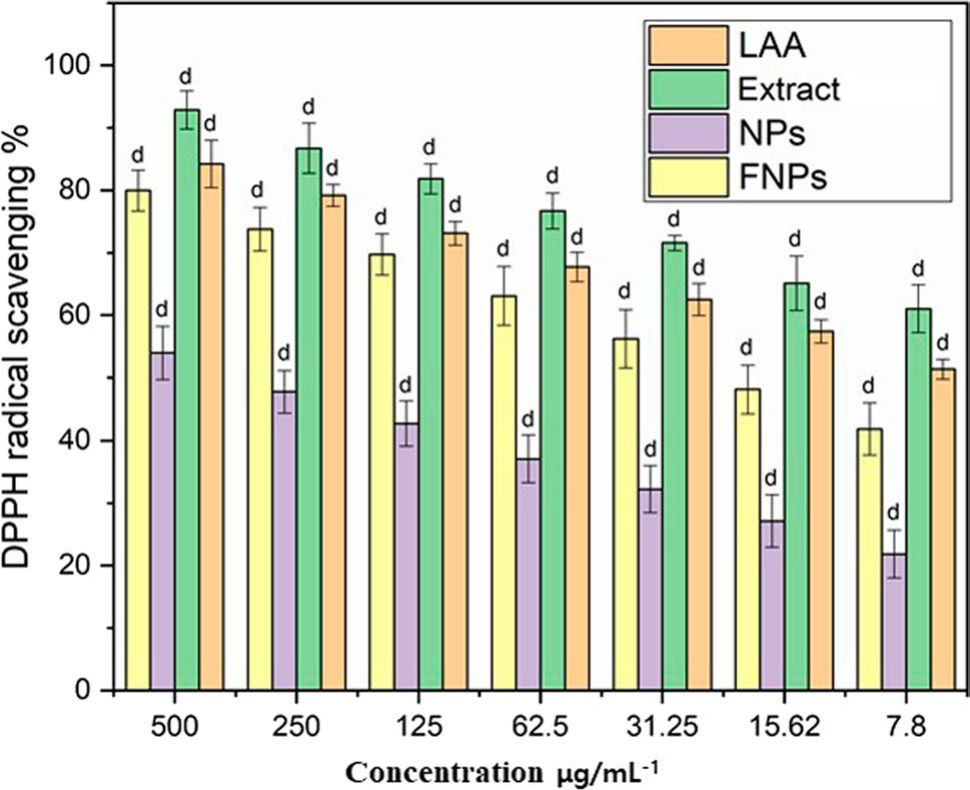
DPPH of control L-Ascorbic Acid (LAA), *M. arvensis* ethanloic extracts (Extract), Nb-N-TiO_2_ (NPs) nanoparticles, functionalized Nb-N-TiO_2_ (FNPs) nanoparticles extracts Error bars represent the standard deviation of the mean and letter d indicate significant difference (*p* < 0.0001)

DPPH (2,2-diphenyl-1-picrylhydrazyl) is a stable compound frequently employed in antioxidant assays due to its specificity and sensitivity to accepting hydrogen or electrons [56]. This investigation evaluated a comparative exploration of the antioxidant capacities within ethanolic leaf extracts of *M. arvensis*, pure Nb-N-TiO_2_, and Nb-N-TiO_2_ functionalized with ethanolic *M. arvensis* extracts [41]. Through a perceptible color alteration, the results underscored the ability and potentiation of functionalized Nb-N-TiO_2_ in DPPH radical scavenging. Ordinarily, solutions containing DPPH exhibit a purple color, yet a transition to a distinctive yellowish color unfolds when they come into contact with antioxidant-rich substances [4]. This chromatic metamorphosis serves as an antioxidant response [8]. The doped Nb-N-TiO_2_, functionalized by the ethanolic extracts of *M. arvensis*, exhibited a substantial DPPH radical scavenging prowess in a concentration-dependent manner. At the concentration of 500 µg mL^−1^, an impressive 79.36% of DPPH free radicals were efficient, near the efficacy of the positive control L-ascorbic acid (LAA), reaching 84.70%. The protocol was validated to process the *M. arvensis* ethanolic extract-mediated Nb-N-TiO_2_ NPs at diverse concentrations with 1 mL of 0.1 mM DPPH suspended in methanol, complemented by 450 µL of 50 mM Tris HCl buffer (pH 7.4) [20]. After a 30-min incubation period, the reduction in DPPH free radicals was quantified by assessing the absorbance at 520 nm [21]. A positive control sample featuring L-Ascorbic Acid (LAA) was the standard control for comparing *M. arvensis* ethanolic extracts, pure doped Nb-N-TiO_2_, and functionalized Nb-N-TiO_2_. The assessment of antioxidant activity spanned multiple diluted concentrations (7.8, 32, 62, 125, 250, and 500 µg mL^−1^), unraveling a spectrum of inhibitory effects spanning from 18 to 90% [22]. Remarkably, doped Nb-N-TiO_2_ NPs functionalized in the company of the plant extract at a concentration of 500 µg mL^−1^ exhibited particularly robust antioxidant activity, achieving an impressive 79% efficacy, in contrast to pure Nb-N-TiO_2_, which attained 53%, as illustrated in Fig. 5 [24].

### Ferric-reducing antioxidant power (FRAP) assay

**Fig. 6:**
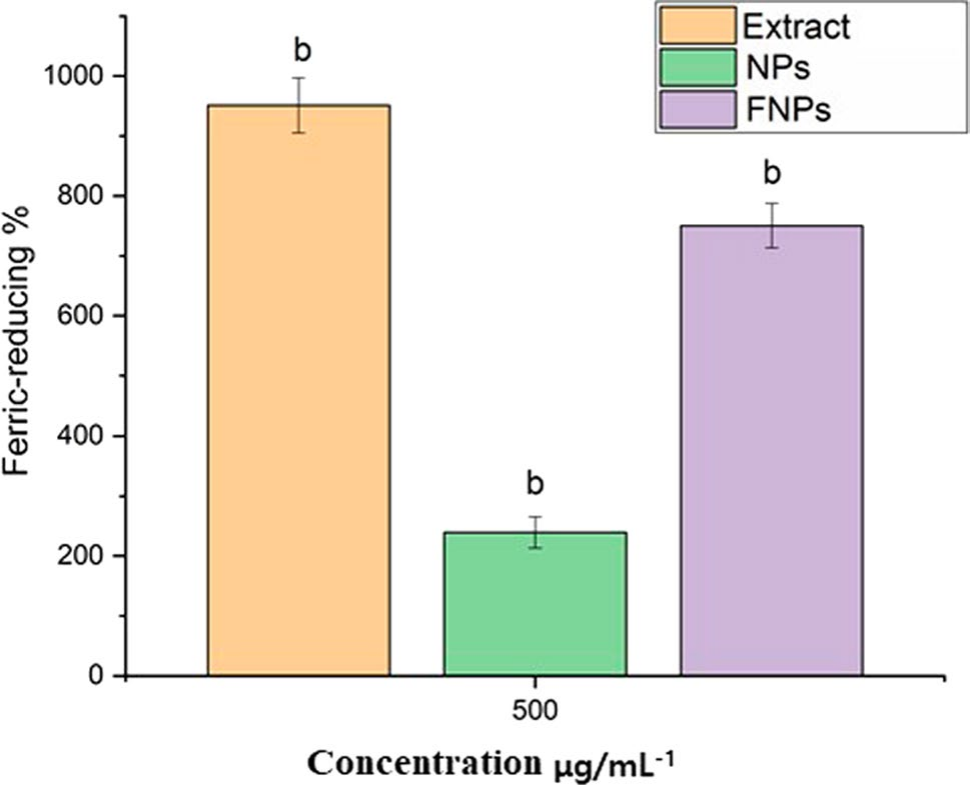
Ferric-reducing antioxidant power (FRAP) of *M. arvensis* ethanloic extracts (Extract), Nb-N-TiO_2_ (NPs) nanoparticles, functionalized Nb-N-TiO_2_ (FNPs) nanoparticles extracts Error bars represent the standard deviation of the mean and letter b indicate significant difference (*p* < 0.001)

The evaluation of ethanolic leaf extracts of *M. arvensis*, pure doped Nb-N-TiO_2_, and Nb-N-TiO_2_ functionalized with *M. arvensis* ethanolic extracts, all displaying the capacity to diminish the Fe^3+^ TPTZ complex effectively [25]. Notably, these reductions approached the efficiency of the positive control, L-ascorbic acid. A standard concentration of 500 µg mL^−1^ was previously used in the FRAP assay; the maximum antioxidant was shown at 500 µg mL^−1^ in the DPPH assay, the ferric reduction efficiency, ethanolic leaf extracts of *M. arvensis*, pure Nb-N-TiO_2_, and the functionalized Nb-N-TiO_2_ exhibited a ferric reduction efficiency of 72.16%, while pure Nb-N-TiO_2_ showed 24.03%, and *M. arvensis* ethanolic extracts achieved 92.9% in 1% potassium ferricyanide, as shown in Fig. 6 [56]. his process of ferric reduction is likely facilitated by the presence of rosmarinic acid and phenolic components that may be enveloping the surface of Nb-N-TiO_2_ synthesized using the ethanolic extracts of *M. arvensis* highlighted that the antioxidant activity of *M. arvensis* extracts, pure Nb-N-TiO_2_, and functionalized Nb-N-TiO_2_ NPs was assessed using the DPPH and FRAP assays, providing a comprehensive evaluation of their antioxidant properties [57].

## Discussion

In this recent research, the functionalizing Nb-N-TiO_2_ NPs explored their potential as antioxidants. This study investigated *M. arvensis* ethanolic extracts, functionalized Nb-N-TiO_2_ with *M. arvensis*-functionalized NPs, and their unmodified pure Nb-N-TiO_2_. This comprehensive exploration also entailed meticulous characterization processes [8]. The outcomes unveiled a remarkable antioxidant activity of functionalized Nb-N-TiO_2_ compared to pure Nb-N-TiO_2_. We employed ethanolic extracts of *M. arvensis* to achieve this functionalization, producing nanoparticles with sizes below 80 nm [26]. The results assessed these nanoparticles' antioxidant efficacy through the DPPH and FRAP assays, affirming their robust antioxidant properties. These findings emphasize the immense potential of functionalized Nb-N-TiO_2_ NPs in biomedical applications, spanning domains such as drug delivery, diagnostics, and therapeutics [48]. This study underscores the substantial promise offered by doped titanium dioxide nanoparticles in various biomedical fields, paving the way for continued research and development to further enhance their utility in diverse biomedical applications [28].

Additionally, another noteworthy study successfully devised an efficient protocol for the functionalization of titanium dioxide nanoparticles using *M. arvensis* ethanolic extracts [42]. This groundbreaking approach opens new avenues for commercializing plant-based nanoparticles, particularly in agriculture, where they can serve as nano-bio-fertilizers and in medicine that underline the viability of plant-based doped nanoparticles as sustainable alternatives across multiple industries [45]. In summary, these studies contribute significantly to the expanding research on doped nanoparticle synthesis with functionalization with plant extracts and biomedical applications, specifically doped Nb-N-TiO_2_ NPs [58]. Functionalized Nb-N-TiO_2_ NPs exhibit various applications across various fields, owing to their unique properties and surface functionalization with M. arvensis extracts. These nanoparticles offer promising opportunities to enhance drug delivery efficiency and minimize side effects in targeted drug delivery systems, and precise targeting capabilities [56]. Similarly, in diagnostics, functionalized NPs can be utilized as contrast agents in imaging techniques like photoacoustic imaging and MRI, facilitating early disease detection by accumulating at disease sites and generating detectable signals [30]. Moreover, in regenerative medicine and tissue engineering, functionalized NPs hold the potential to promote cell growth, proliferation, and differentiation, thereby aiding tissue regeneration processes through surface functionalization with bioactive molecules from *M. arvensis* extracts. Furthermore, nano-bio-fertilizers present significant prospects for agriculture, utilizing functionalized NPs derived from plant extracts to enhance crop productivity, nutrient uptake, and soil health [55]. Case studies have demonstrated the successful use of such nano-bio-fertilizers in various agricultural settings, contributing to improved crop growth and yield [21]. Moreover, adopting plant-based extracts for nanoparticle functionalization aligns with principles of green chemistry and sustainability, offering environmental benefits such as reduced reliance on traditional chemical synthesis methods and decreased environmental pollution [3]. Overall, plant-based nanoparticle synthesis methods' scalability and economic feasibility underscore their importance in sustainable nanoparticle synthesis practices, further highlighting the potential of functionalized Nb-N-TiO_2_ NPs in diverse applications [47]. These functionalized NPs can play pivotal roles in drug delivery systems and diagnostics [32]. Furthermore, these investigations highlight the importance of exploring doped nanoparticles and their functionalization with plant-based extracts for efficient and sustainable nanoparticle synthesis and application methodologies [59].

## Conclusion

This study has achieved a significant milestone by successfully functionalizing doped Nb-N-TiO_2_ NPs using ethanolic extracts derived from *M. arvensis* leaves. First, characterization analysis was performed using FTIR, SEM, and UV–Vis spectrophotometry techniques, and then extensive antioxidant assays were conducted to assess their potential, revealing their exceptional antioxidant properties. Significantly, these functionalized doped nanoparticles displayed heightened effectiveness compared to their pure Nb-N-TiO_2_ NPs. These findings underscore the promising applications of *M. arvensis*-mediated Nb-N-TiO_2_ NPs in biomedicine, particularly as antioxidants. Their enhanced efficacy and reduced toxicity positions them as highly prospective candidates for various biomedical purposes, including drug delivery systems, diagnostics, and therapeutic interventions.

Furthermore, developing functionalized doped Nb-N-TiO_2_ NPs using *M. arvensis* highlights the versatility of plant-based NPs as sustainable alternatives; this extends their potential utility into sectors such as medicine, where they can serve as nano-bio-medicine and in diverse biomedical applications. Collectively, these research endeavors make substantial contributions to the burgeoning field of doped nanoparticles with functionalized plant extracts, with a specific focus on doped titanium dioxide nanoparticles. They underscore the essence of embracing sustainable methodologies throughout the synthesis and utilization of these doped functionalized nanoparticles across medical industries.

## Data Availability

The data will be provided on reasonable request.
